# Haemoglobin level increase as an efficacy biomarker during axitinib treatment for metastatic renal cell carcinoma: a retrospective study

**DOI:** 10.1186/s12885-017-3312-7

**Published:** 2017-05-22

**Authors:** Alison C. Johnson, Margarida Matias, Helen Boyle, Bernard Escudier, Alicia Molinier, Brigitte Laguerre, Carole Helissey, Pierre-Emmanuel Brachet, Audrey Emmanuelle Dugué, Loic Mourey, Elodie Coquan, Florence Joly

**Affiliations:** 1 Centre François Baclesse, F-14000 Caen, France; 2 Institut Gustave Roussy, F-94800 Villejuif, France; 3 Centre Léon Bérard, F-69008 Lyon, France; 4 Institut Claudius Regaud, F-31000 Toulouse, France; 5 Centre Eugène Marquis, F-35000 Rennes, France; 6 HIA Bégin, F-94160 Saint-Mandé, France

**Keywords:** Axitinib, Haemoglobin, High blood pressure, Polycythemia, Prognosis, Renal cell carcinoma

## Abstract

**Background:**

Axitinib is used after failure of first line treatment for metastatic renal cell carcinoma (mRCC). A known side effect is the increase of haemoglobin level (HbL) during treatment with a suspected correlation with better outcome. Our objective was to examine whether HbL increase during the first three months of axitinib treatment is associated with better prognosis.

**Methods:**

Retrospective multicentre analysis including patients with mRCC treated with axitinib for at least three months from 2012 to 2014. Progression-free survival (PFS) was analysed by a Cox model according to gender, International Metastatic Renal Cell Carcinoma Database Consortium (IMDC) prognostic score, high blood pressure (hBP), and maximum increase in HbL within the first three months of treatment.

**Results:**

Ninety-eight patients were analysed (71% men; median age at treatment initiation: 62 years; IMDC: 24%, 50%, and 26% in the favourable, intermediate, and poor-risk group, respectively). Patients received axitinib for a median of 8 months. During the first three months, the median increase of HbL was +2.3 g/dL (−1.1; 7.2). Fifty-six (57%) patients developed hBP.

In multivariate analysis, after adjustment for performance status (*P* < 0.0001) and gender (*P* = 0.0041), the combination of HbL increase ≥2.3 g/dL and any grade hBP was significantly associated with longer PFS (HR = 0.40, 95%CI [0.24; 0.68]).

**Conclusions:**

Early HbL increase during axitinib treatment combined with hBP is an independent predictive factor of PFS. These results require validation in a prospective setting.

## Background

Renal cancer represents 2–3% of all cancers, with an increased incidence in Western countries. The most common form is renal cell carcinoma (RCC) and approximately 30% of patients will present metastatic disease (mRCC) [1]. Better insight into the molecular pathways involved in RCC has spurred the development of novel targeted therapies. One such pathway involves loss of function of the von Hippel-Lindau (VHL) tumour-suppressor gene leading to vascular endothelial growth factor (VEGF) overexpression, which promotes neo-angiogenesis [2]. Molecular agents targeting angiogenesis, such as anti-VEGF monoclonal antibodies and tyrosine kinase inhibitors (TKI) acting on the VEGF receptor (VEGFR), have become a standard of care in mRCC.

The TKI axitinib is an oral, potent, and selective VEGFR-1, −2, and −3 inhibitor, used after failure of a prior first-line treatment with cytokines or sunitinib for the treatment of mRCC. Common side effects associated with axitinib are high blood pressure (hBP), diarrhoea, fatigue, decreased appetite, nausea, and dysphonia [3, 4]. Studies have shown that some adverse effects, such as the onset of hBP, are correlated to treatment efficacy [5, 6].

In the phase III study AXIS, which compared axitinib (*n* = 361) with sorafenib (*n* = 362) as second-line therapy in 723 patients with mRCC, 10% of patients treated with axitinib presented elevated haemoglobin, requiring phlebotomy in three patients. Several other cases of early haemoglobin level increase during various antiangiogenic treatments have been reported since. These increases appeared a few weeks after treatment initiation and seemed associated with better outcomes [7–11].

Based on these observations, we performed a retrospective analysis to determine whether early haemoglobin level increase during axitinib treatment in mRCC is associated with better prognosis.

## Methods

### Study design and patients

This was a retrospective multicentre study. Patients 18 years or older, with histologically confirmed metastatic RCC, treated with axitinib for at least three months, initiated from 2012 to 2014 in six French cancer centres by physicians belonging to the French genitourinary tumour study group (GETUG), were included. Patients with prior polycythaemia and those who received a blood transfusion during the first three months of axitinib were excluded. There were no limitations on the number of previous lines of treatment. Data were collected from clinical and radiological files and recorded by the same investigator using a standardized form.

In accordance with local laws, this study was approved by a national ethical committee and a local institutional review board.

### Studied parameters and definitions

Biological parameters were recorded before and during axitinib treatment. We analysed haemoglobin changes during the first three months of axitinib and our main criterion was the maximal HbL increase, dichotomized using the median value.

Cut-offs for polycythaemia were chosen based on revised World Health Organization diagnostic criteria [12]. Polycythaemia was defined as haematocrit above 56% or haemoglobin level (HbL) above 16.5 g/dL in females and haematocrit above 60% or HbL above 18.5 g/dL in males, or HbL superior to 17 g/dL in men and 15 g/dL in women with a sustained increase ≥2 g/dL from baseline, in the absence of iron deficiency treatment or hemo-concentration.

Adverse events (AE) were graded using the National Cancer Institute Common Terminology Criteria for Adverse Events (NCI CTCAE) version 4.0 [13].

We applied the International Metastatic Renal Cell Carcinoma Database Consortium (IMDC) model at baseline based on six risk factors: Karnofsky performance status <80%; serum calcium, platelet count, and neutrophil count above upper limit of normal; HbL below lower limit of normal; and time from initial diagnosis to treatment initiation <1 year [14, 15]. Patients with no prognostic factors were favourable-risk, those with one or two were intermediate-risk, and those with more than two were poor-risk.

Radiological evaluations were extracted from patient files. Objective response rate (ORR) was defined as the proportion of patients with partial or complete response by investigator assessment. Efficacy measures analysed were progression-free survival (PFS) and overall survival (OS). PFS was defined as the time from axitinib initiation to first documentation of disease progression or death whichever came first. OS was defined as the time from treatment initiation to death from any cause. At the last follow-up, patients with no events (progression and/or death), were censored for PFS and OS, respectively.

### Statistical analysis

Categorical variables were described as frequencies and percentages and continuous variables as medians and ranges. Comparisons between groups were done using Mann-Whitney test for continuous variables and Chi-squared or Fisher’s exact test for binary variables, as appropriate.

PFS and OS were estimated over time using the Kaplan–Meier method; continuous variables were dichotomized using their median value. PFS was compared between groups using log-rank test. For PFS multivariate analysis, a Cox proportional hazards model was used, including parameters achieving *P* value ≤0.20 in univariate analysis. Variable collinearity was checked before multivariate computation in order to put only independent PFS predictors in the model. A composite variable could be computed in case of collinearity. Akaike Information Criterion (AIC) was used to select the most parsimonious multivariate model.

OS was stratified for treatment line (2nd-3rd line vs. beyond), comparisons between groups were done with stratified log-rank test. For OS multivariate analysis, the same procedure as for PFS multivariate analysis was followed but using a stratified Cox proportional hazards model. All analyses were performed with R software, version 3.1.2 (R Foundation for Statistical Computing).

## Results

### Patient characteristics

**Fig. 1:**
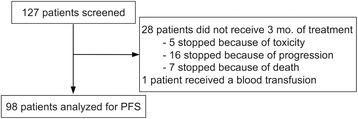
Flow-chart of study population

Information was collected in six French cancer centres for 127 patients with metastatic renal-cell carcinoma treated with axitinib who met eligibility criteria (Fig. 1). The efficacy analysis was conducted among the 98 patients who had received axitinib for at least three months. Their characteristics are described in Table 1.

**Table 1:** Baseline characteristics and treatment modalities

Characteristics	Total (*n* = 98)
Demographics:	
Male	70 (71)
Age at treatment start (yr.)	62 [24–82]
IMDC score at treatment start (*n* = 80)	
Favourable risk	19 (24)
Intermediate risk	40 (50)
Poor risk	21 (26)
Medical history:	
Hypertension (*n* = 73)	14 (19)
Tobacco use (*n* = 86)	
Active	19 (22)
Ceased	24 (28)
None	43 (50)
Tumour characteristics:	
Histology	
Clear cell	85 (87)
Other^a^	13 (13)
Fuhrman grade (*n* = 79)	
I-II	23 (29)
III	40 (51)
IV	16 (20)
TNM staging (*n* = 80)	
T1	15 (19)
T2	21 (26)
T3–4	44 (55)
M1 at initial diagnosis	36 (45)
Pulmonary metastasis (*n* = 98)	77 (78)
Treatment:	
Prior nephrectomy	86 (88)
Number of lines of treatment at axitinib start	3 [2–7]
Treatment duration (mo.)	8 [3–30]
Causes of axitinib discontinuation (*n* = 83)	
Progression	57 (68.5)
Toxicity	13 (15.5)
Death	9 (11)
Other	4 (5)
Biology at axitinib start:	
Haemoglobin serum level (g/dL) (*n* = 98)	12.5 [8.4–16.8]
Creatinine serum level (μmol/L) (*n* = 78)	101.5 [39–215]
Chronic kidney disease (*n* = 78)	
Grade 1	41 (52)
Grade 2	36 (46)
Grade 3	1 (1)

^a^Other histology was papillary (*n* = 7), juvenile or Xp11 translocation RCC (*n* = 4), chromophobe (*n* = 1), and sarcomatoid (*n* = 1)

### Axitinib treatment

Treatment modalities are presented in Table 1. Axitinib was administered as 2nd or 3rd line treatment in 67 (68%) patients; 90% of patients had received sunitinib prior to axitinib. According to the IMDC model, 74% of patients were in the favourable or intermediate risk groups. At the time of analysis, median axitinib treatment duration was 8 months (range 3–30) and 15 (15%) patients were still on treatment. Patients received axitinib initially at 5 mg twice daily (bid) and the dose was increased according to axitinib label to 7 mg bid and 10 mg bid in 28 (29%) and 20 (20%) patients, respectively. Three (3%) patients started with doses of 3 mg bid due to frailty or residual side effects from previous therapy. No hematopoietic growth factors were used. The most common reason for treatment discontinuation was disease progression (68.5%).

### Overall efficacy

Among patients treated for at least three months, 28 (29%) and 2 patients presented partial and complete response, respectively. ORR was 31% (30/98). After a median follow-up of 16.3 months from axitinib initiation (range 2.6; 34.1), median PFS and OS were 9.0 (95%CI 7.4; 11.3) and 23.4 (95%CI 19.4; not reached) months, respectively.

### Overall safety during axitinib treatment

Sixty (62%) patients presented grade 2 toxicities and 59 (61%) presented grade 3 toxicities. No grade 4 toxicities were observed. The three most common AEs (fatigue, hBP, and diarrhoea) were present in more than 50% of patients (Table 2).

**Table 2:** Adverse events during axitinib treatment

	Total (*n* = 98)		HbL increase <2.3 g/dL (*n* = 49)		HbL increase ≥2.3 g/dL (*n* = 49)		*p*
	All grades	Grade III	All grades	Grade III	All grades	Grade III	
Fatigue	61 (62)	19 (19)	30 (61)	11 (22)	30 (61)	8 (16)	1.00
Arterial hypertension	56 (57)	32 (33)	21 (43)	16 (33)	35 (71)	16 (33)	0.0043
Diarrhoea	55 (56)	11 (11)	23 (47)	2 (4)	31 (63)	9 (18)	0.10
Dysphonia	36 (37)	1 (1)	14 (29)	1 (2)	22 (45)	0	0.09
Hand-foot syndrome	23 (23)	9 (9)	10 (20)	5 (10)	13 (26.5)	4 (8)	0.47
Anaemia	23 (23)	2 (2)	15 (31)	1 (2)	8 (16)	1 (2)	0.15^*^
Musculo-skeletal pain/arthralgia	19 (19)	2 (2)	7 (14)	1 (2)	12 (24)	1 (2)	0.31^*^
Mucositis	17 (17)	3 (3)	4 (8)	1 (2)	13 (26.5)	2 (4)	0.03^*^
Anorexia	17 (17)	4 (4)	6 (12)	1 (2)	11 (22)	3 (6)	0.29^*^
Hypothyroidism	11 (11)	0	5 (10)	0	6 (12)	0	1.00^*^
Nausea/vomiting	8 (8)	1 (1)	4 (8)	0	4 (8)	1 (2)	1.00^*^
Rash	5 (5)	1 (1)	2 (4)	0	3 (6)	1 (2)	1.00^*^
Proteinuria	2 (2)	2 (2)	1 (2)	1 (2)	1 (2)	1 (2)	1.00^*^

^*^ Fisher’s exact test; all other results obtained with chi-square test

### Evolution of haemoglobin levels and polycythaemia

**Fig. 2:**
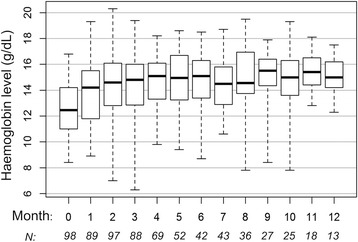
Haemoglobin level (g/dL) during the first 12 months of axitinib treatment. Boxplots represent quartiles and extreme values

HbL during axitinib treatment is described in Fig. 2. Half (*n* = 50) of all patients had an HbL below the lower limit of normal (LLN) at treatment initiation: 37 presented grade 1 anaemia (HbL 10 g/dL - LLN) and 13 presented grade 2 anaemia (HbL 8-10 g/dL). During the first three months of treatment, median maximum HbL increase was +2.3 g/dL (range − 1.1; +7.2). HbL increased 2 to 3 g/dL in 23 (23%) patients and more than 3 g/dL in 32 (33%) patients. Thirteen of the 49 (27%) patients with HbL increase ≥2.3 g/dL achieved objective partial response in the first three months of axitinib, 33 (67%) were considered stable, and 3 (6%) progressed. Of the 33 patients with stable disease, two patients achieved a later partial response, after five and six months of treatment, respectively.

According to the WHO criteria previously defined, 16 patients (16%) presented polycythaemia during the first three months. All of them also presented any grade hBP. Four patients had clinical symptoms including headaches, facial erythema, and concomitant thromboembolic events requiring anticoagulation therapy. Four patients were treated by phlebotomy; axitinib dose was reduced in three. Patients with polycythaemia had a higher median baseline HbL than those without (14, range 9.1; 16.5 vs. 12.3, range 8.4; 16.8 g/dL; *P* value = 0.018).

### Parameters linked to HbL increase

While comparing patients with and without HbL increase ≥2.3 g/dL, we did not observe any differences regarding age, TNM staging, IMDC score, initial haemoglobin levels or axitinib dose at time of maximal Hb increase. There were however significantly more males (40/49 vs. 30/49; *P* value = 0.025) and lower Fuhrman grades (*P* value = 0.0013) in the group with HbL increase ≥ + 2.3 g/dL. Differences regarding AEs between the two groups are described in Table 2. Treatment duration was significantly different between these two groups with a median of 11 months (range 3; 30) for patients with an HbL increase ≥ + 2.3 g/dL vs. 7 months (range 3; 23) for those without; *P* value = 0.013.

### Factors associated with survival

**Fig. 3:**
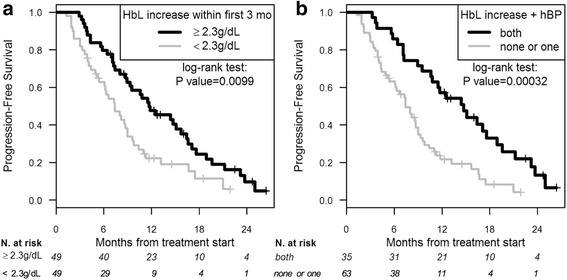
PFS in patients with and without HbL increase ≥2.3 g/dL during the first three months of axitinib treatment (**a**). PFS in patients with HbL increase ≥2.3 g/dL combined with hBP and with either or no factors (**b**). HbL: haemoglobin level; hBP: high blood pressure; mo.: months

Factors associated with PFS in univariate analysis are summarized in Table 3. Patients with an HbL increase during the first three months of treatment ≥2.3 g/dL had significantly longer PFS than those without such increase (median PFS of 11.7 vs. 7.4 months, respectively; *P* value = 0.0099) (Fig. 3a). No significant difference in PFS was detected between patients who presented polycythaemia during the first three months and those who did not (median of 10.5 vs. 8.9 months; *P* value = 0.53). As expected, any grade hBP was also predictive of longer PFS (median PFS of 11.2 vs. 7.3 months; *P* value = 0.0047).

**Table 3:** Univariate and multivariate PFS analyses. Univariate *P* values were computed by the log-rank test, multivariate *P* values by the Cox proportional hazards model

	Univariate analysis (*N* = 98)				Multivariate analysis (*N* = 96)		
	*N*	median PFS	95%CI	*P* value	adjusted HR	95%CI	*P* value
Demographics							
Gender				0.016			0.0041
Male	70	10.4	[8.6; 14.7]		1	-	
Female	28	7.3	|6.1; 11.0]		2.1	[1.3; 3.5]	
Age				0.19			
< median (61.6 years)	49	8.0	[6.6; 10.8]				
≥ median (61.6 years)	49	11.0	[8.2; 17.1]				
Smoking status				0.61			
Never	43	8.7	[7.2; 15.1]				
Former	24	8.7	[5.6; NR]				
Current	19	8.2	[4.1; 10.6]				
Disease characteristics							
Histology				0.17			
Clear cell carcinoma	85	9.5	[8.0; 11.8]				
Other	13	5.6	[4.0; NR]				
Fuhrman grades				0.29			
I-II	23	11.5	[8.7; 17.6]				
III	40	8.4	[6.4; 11.3]				
IV	16	13.2	[9.0; NR]				
Nephrectomy				0.63			
No	12	6.1	[4.3; NR]				
Yes	86	9.3	[8.0; 11.7]				
Axitinib treatment							
Treatment line				0.19			
2nd-3rd line	67	9.5	[8.0; 13.2]				
4th line and beyond	31	7.4	[5.4; 14.7]				
Baseline IMDC				0.0026			
Favourable risk	19	11.5	[7.4; NR]				
Intermediate risk	40	11.8	[9.0; 16.7]				
Poor risk	21	6.3	[3.8; 9.3]				
Performance status				0.00044			<0.0001
0	24	15.7	[11.3; NR]		1	-	
1	54	8.7	[7.2; 11.7]		2.4	[1.3; 4.4]	
2–3	18	6.3	[3.6; 9.6]		5.5	[2.5; 11.9]	
Anaemia							
No	48	8.8	[8.7; 13.1]	0.34			
Yes	50	7.7	[7.4; 10.6]				
BMI				0.38			
< median (24.8 kg/m^2^)	42	9.0	[7.1; 11.8]				
≥ median (24.8 kg/m^2^)	43	8.9	[6.6; 14.4]				
Adverse events							
HbL increase *(during first 3 mo.)*				0.0099			
< median (2.3 g/dL)	49	7.4	[6.1; 9.6]				
≥ median (2.3 g/dL)	49	11.7	[9.3; 16.6]				
hBP *(during treatment)*				0.0047			
No	42	7.3	[4.4; 9.3]				
Any grade	56	11.2	[8.9; 16.4]				
Composite factor:							
HbL increase ≥2.3 g/dL and hBP				0.00032			0.00048
None or only one	63	7.4	[6.2; 9.0]		1	-	
Both	35	14.7	[10.8; 19.6]		0.40	[0.24; 0.68]	

*NR* not reached, *95%CI* 95% confidence interval, *IMDC* International Metastatic Renal Cell Carcinoma Database Consortium, *BMI* body mass index, *hBP* high blood pressure, *HbL* haemoglobin level

HbL increase (≥ 2.3 g/dL) and any grade hBP were collinear and could therefore not be inserted in the same model, we thus computed a composite criterion with both factors: patients with HbL increase and hBP had significantly longer PFS than those with only one of these factors or neither (median PFS 14.7 months vs. 7.4 months, *P* value = 0.00032) (Fig. 3b).

For multivariate analysis, we studied the composite criterion of hBP and HbL increase, which was the strongest predictor of PFS in univariate analysis. As described in Table 3, after adjustment for performance status (*P* value <0.0001) and gender (*P* value = 0.0041), the presence of both HbL increase ≥2.3 g/dL and any grade hBP was an independent predictor of PFS, with an HR of 0.40 (95%CI 0.24; 0.68; *P* value = 0.00048). Using AIC, the multivariate model taking into account this composite factor was better than the one obtained with either factor individually.

Concerning OS, in the univariate analysis, clear cell histology, better performance status or more favourable IMDC group, nephrectomy, any grade hBP, older age at treatment initiation, higher BMI, and the presence of both HbL increase ≥2.3 g/dL and hBP were associated with a better OS, after stratifying for treatment line. In the multivariate analysis, after adjustment for performance status (*P* value = 0.00031) and stratifying for treatment line, any grade hBP remained the best independent predictor of longer OS with an HR of 0.40, 95%CI 0.22; 0.75, *P* value = 0.0038 (data not shown).

## Discussion

Our study suggests that an increase in haemoglobin level in the first three months of axitinib treatment is associated with longer PFS, implying that such increases could be an early indicator of drug activity. When combining HbL increase with hBP, this association becomes a stronger predictor of PFS than either factor alone. HbL increase could thus be an additional early biomarker of treatment efficacy, complementary to radiological evaluations, and appearing in most cases before the radiological evaluations which are typically performed at three months of treatment. Two of our patients with early HbL increase presented delayed partial responses to axitinib, suggesting axitinib be continued in cases with early HbL increase, in the absence of dose-limiting toxicities or progressive disease.

To our knowledge, this is the largest retrospective study of axitinib-induced haemoglobin changes. High BP is already known as a marker of axitinib efficacy [6], but the fact that haemoglobin elevation could be a simple, early efficacy biomarker predictive of outcome is novel. These findings are in agreement with previous published cases reporting increased HbL associated with several VEGF inhibitors, including bevacizumab, sunitinib, sorafenib, and axitinib [7–11]. In most cases, erythropoiesis developed in the first month after treatment initiation and was reversible at treatment discontinuation. Some of these cases report transient erythropoietin (EPO) increases [10, 11]. Bhatta et al. retrospectively analysed several trials with VEGF inhibitors and found a correlation between VEGF inhibitor exposure, increased EPO, and red blood cell counts, independently of blood pressure or creatinine clearance changes [16]. Although the physiopathology of this haemoglobin increase is not yet fully understood, EPO is likely to play a part. A hypothesis explaining axitinib-related haemoglobin elevation is that VEGF blockage induces rebound erythrocytosis by stimulating hypoxia-inducible factors such as EPO. Tam et al. found that VEGFR-2 inhibition by aflibercept in animal models lead to hepatic EPO production and erythrocytosis detectable after 4 weeks of treatment [17]. In cases with normal or diminished EPO levels, authors suggested increased sensitivity to EPO due to TKI treatment as a possible mechanism [8]. Many disease-related factors such as inflammation, tumour bleeding or malnutrition may have favoured anaemia in some of our patients. Axitinib could have corrected anaemia by decreasing tumour volume, thus leading to HbL increase. It is unlikely that the increase in HbL was paraneoplastic as it was temporally related with treatment administration and associated with better objective responses and longer PFS. EPO levels, VHL mutational status, and JAK2V617F status were not available for most patients in this study.

Polycythaemia, defined by revised WHO criteria [12], was not correlated to outcome, although the small number of cases limits statistical power. The polycythaemia criteria were perhaps too stringent. The gold standard for diagnosis is isotopic red cell mass measurement (RCM) and there is debate over whether haemoglobin or haematocrit levels alone can substitute for RCM. In the polycythaemia group, baseline haemoglobin before axitinib initiation was higher and this has been shown to be a favourable prognostic factor [18]. Also, polycythaemia management was not standardized with dose reduction for three patients while others continued on full-dose therapy, which could account for differences in outcome. We cannot draw definitive guidelines for polycythaemia management on axitinib treatment from this analysis, but our results encourage us to continue axitinib with concomitant symptomatic treatment.

The PFS observed in our study are not comparable to those of the main phase III axitinib study (AXIS) as we excluded patients who had received less than three months of treatment, thus removing from our analysis many patients with short PFS [4]. Interestingly, 31% of patients received axitinib beyond the 3rd line of treatment, a situation for which very few data are available.

Our study was limited by its retrospective design, precluding complete data collection, but this was compensated by standardized data collection, patient referral through the GETUG network, and rigorous selection criteria, with the exclusion of patients with insufficient data.

The use of the median as an arbitrary cut-point for the study of the continuous variable HbL, while allowing for a simple, practical biological threshold, reduces statistical power [19].

We cannot control for the possibility that anaemia present at baseline in certain patients was due to prior therapy and that haemoglobin normalization during axitinib treatment was only due to washout of the previous treatment.

The absence of impact of HbL increase alone on OS could be due to following lines of treatment, which were not controlled for. Occurrence of hBP in patients with HbL increase raises questions about the complementary link between both factors. Some authors suggest the two are correlated and due to a decrease in nitric oxide production by VEGFR inhibition, leading to loss of plasma volume [10]. This may explain why both factors together were associated with better outcomes than each factor considered individually.

## Conclusion

This retrospective study suggests that early haemoglobin level increase during axitinib treatment in patients with metastatic renal cell carcinoma is associated with significantly improved clinical outcome. When combined with elevated blood pressure, it is a stronger predictive factor of better outcome than either factor considered separately. This easily manageable and measurable potential adverse event biomarker requires prospective validation.

## Abbreviations

AE: Adverse events
AIC: Akaike information criterion
EPO: Erythropoietin
HbL: Haemoglobin level
hBP: High blood pressure
IMDC: International metastatic renal cell carcinoma database consortium
mRCC: Metastatic renal cell carcinoma
ORR: Objective response rate
OS: Overall survival
PFS: Progression-free survival
RCM: Red cell mass measurement
TKI: Tyrosine kinase inhibitors
VEGF: Vascular endothelial growth factor
VEGFR: Vascular endothelial growth factor receptor
VHL: von Hippel-Lindau
